# Utilization of Waste Rubber Materials After the End of Their Life Cycle in the Production of Three-Layer Particleboards—Physical and Mechanical Properties

**DOI:** 10.3390/polym17070998

**Published:** 2025-04-07

**Authors:** Vladimír Mancel, Iveta Čabalová, Jozef Krilek, Çağrı Olgun, Mustafa Öncel, Önder Tor, Tomasz Szul, Grzegorz Woroniak, Joanna Piotrowska-Woroniak

**Affiliations:** 1 Department of Environmental and Forestry Machinery, Faculty of Technology, Technical University in Zvolen, Študentská 26, 96001 Zvolen, Slovakia; krilek@tuzvo.sk; 2 Department of Chemistry and Chemical Technologies, Faculty of Wood Sciences and Technology, Technical University in Zvolen, T. G. Masaryka 24, 96001 Zvolen, Slovakia; cabalova@tuzvo.sk; 3 Department of Forest Industry Engineering, Faculty of Forestry, Kastamonu University, Orgeneral Atilla Ateş Paşa Cd., 37210 Kastamonu, Turkey; colgun@kastamonu.edu.tr (Ç.O.); moncel@kastamonu.edu.tr (M.Ö.); ondertor@kastamonu.edu.tr (Ö.T.); 4 Department of Bioprocess Engineering, Energy and Automation, Faculty of Production and Power Engineering, University of Agriculture in Krakow, Balicka 116 B, 30149 Krakow, Poland; t.szul@urk.edu.pl; 5 Department of HVAC Engineering, Faculty of Civil and Environmental Sciences, Bialystok University of Technology, Wiejska 45E, 15351 Bialystok, Poland; g.woroniak@pb.edu.pl (G.W.); j.piotrowska@pb.edu.pl (J.P.-W.)

**Keywords:** three-layer particleboard, wood particles, waste rubber, life cycle

## Abstract

The aim of the article was to test new types of rubber-containing particleboards created from waste materials, which positively contributes to environmental protection, saving primary resources and reducing production costs. This article focuses on the study of three-layer particleboards made from wood particles (spruce non-treated beams) and waste rubber granulates (tires, mixture of seals and carpets, internal flammable cables, external non-flammable cables). Urea–formaldehyde glue, melamine–formaldehyde glue, paraffin emulsion, and ammonium nitrate were used as a binders and excipients in the manufacturing of particleboards. In the core layer of each particleboard, 10% of the weight was made up of rubber granulate. Physical properties (density, water absorption, thickness swelling) and mechanical properties (internal bonding strength, modulus of rupture, modulus of elasticity, screw driving torque) were assessed from this perspective using current EN technical standards. According to the findings, the average densities of all particleboards were comparable to each other in a range from 0.692 to 0.704 g·cm^−3^. The lowest average water absorption and thickness swelling reached particleboards containing 10% of waste internal flammable cables, namely 32.79% for water absorption and 13.21% for thickness swelling. The highest average internal bonding strength reached particleboards without rubber filler and particleboards containing 10% of waste external non-flammable cables, namely 0.52 MPa for both types. The highest average modulus of rupture reached particleboards without rubber filler, namely 12.44 MPa. The highest average modulus of elasticity reached particleboards containing 10% of waste internal flammable cables, namely 2206.29 MPa, and the highest screw driving torque reached particleboards without rubber filler, namely 0.46 N·m for seating torque and 1.44 N·m for stripping torque. The results show that particleboards containing waste external non-flammable cables and particleboards containing waste internal flammable cables achieved comparable results to particleboards without rubber filler, which provides a good basis for a new way of utilizing this type of waste in the form of producing new wood–rubber composites.

## 1. Introduction

The content of created rubber trash has increased as a result of the industry’s ongoing expansion and consumer lifestyles. Due to its sluggish rate of degradation, this waste is persistent in the environment. There is a steady rise in the amount of garbage based on rubber also because there are more and more cars on the roads. According to Dwivedi et al. [1], when all tire kinds are included, the yearly tire production rate worldwide is 1400 million units, with an estimated 17 million tons of old tires produced annually. Worn tires and other types of waste rubber from cars, such as seals, carpets, and cables, are a major issue on a global scale. The composition of this type of garbage is still far more than the amount that can be evaluated logically. By 2030, 1200 million tons of tires are expected to be thrown annually [2]. Synthetic polymers, such as polyamide, butyl rubber, butadiene rubber, and styrene butadiene rubber, make up 46–48% of the material in tires [3]. Rubber is given carbon black to increase abrasion resistance. The second important component of tires is silica, which is combined with carbon black [4]. The most common components of tires, according to the study by Danon and Gorgens [5], are natural rubber (14–27%), synthetic rubber (14–27%), and fillers such as silica (26–28%), sulfur (5–6%), plasticizers derived from oils and resins (5–6%), steel and textile wires, etc. Automotive seals are typically dual extrusion materials of sponge and dense rubber that are attached to either the door or the car body in order to seal the passenger compartment [6]. Automotive carpet is a woven composite material manufactured from a variety of materials. Many modern carpets and carpet padding are of petrochemical origin [7]. In addition, 80% of automotive carpets consist of PET, polyethylene (PE) films, and composite materials. In rubber material production, natural rubber (NR), isoprene rubber (IR), nitrile butadiene rubber (NBR), styrene butadiene rubber (SBR), and ethylene propylene diene monomer rubber (EPDM) are most commonly used [8].

There are not many possibilities for obtaining original-quality rubber or other comparable rubber-based materials. Tires can be used as flooring, noise barriers, energy recovery, and other secondary applications. Rubber can be recycled in one way, by being ground into a powder and then combined with thermoplastic resins. Thermoplastic elastomer compounds, which are multipurpose polymer materials, are created by this procedure. Their flexibility and ease of processing are advantageous qualities [9]. Utilizing rubber waste as a component for composites processing is another approach to value rubber waste. Composite materials made from natural (wood) and synthetic polymers (rubber/plastic) have several positive qualities. Such composites can be utilized in the infrastructure, transportation, and building sectors [10]. At a high temperature, between 170 °C and 200 °C, wood particles are combined with a synthetic polymer to create composite materials. Some issues can arise at these high temperatures, such as the extractive chemicals from wood migrating to the surface. This can be the result of a decline in the mechanical properties of the composites as well as interactions between the synthetic polymer, the wood, and the used binder being reduced [11,12,13,14,15]. Wood particles and rubber granulates have significantly different properties. Wood particles have different strengths and elastic modulus. The properties of composite materials strongly influence the flexural strength properties, their volume ratio, and the interaction between them from the morphological point of view of each component [16]. Since rubber and wood have different surface properties where rubber is hydrophobic while wood is hydrophilic, surface treatment of waste rubber materials is required before bonding with wood. Methods include pre-treatment with coupling agents such as titanate [17], silane [18], and mechanical grinding or rolling of rubber materials [17], which help to improve the interfacial bonding between rubber materials and wood particles [19]. The weight percentage of the components, the kind of additives, the pressing temperature, etc., all affect the mechanical properties of composites [20]. A synthetic polymer can be added to the wooden matrix to enhance some of the physical characteristics of particleboards (PBs). Ayrilmis et al. [16] claim that increasing the rubber crumb/wood particle ratio enhances the boards’ water resistance. Finding the best circumstances for the creation of composites, such as pressing duration and temperature, resin content, board density, etc., is preferable for producing wood-based composites [21].

One of the earliest building materials is wood. Wood is a material that originates from renewable resources, is easily machined, and has a high strength-to-weight ratio. Although wood has a long history of usage in construction and has many environmental advantages, its interaction with water makes it challenging to employ as a building material. Water alters the dimensions of wood, which is one of the main problems with using it. Wood readily takes up moisture from the surroundings. Swelling results from this absorption, while contraction follows from desorption [22]. Wood composites are important for many structural and non-structural applications for indoor and outdoor uses, including furniture, structural components, flooring, windows, and doors. They also have many uses in the building, infrastructure, and transportation industries [10,23]. Due to its strong adhesion, cohesiveness, and affordable pricing, urea–formaldehyde (UF) resin is frequently used to create wood composites. Although PB has several benefits, the three main drawbacks are water absorption, flammability, and formaldehyde emissions. Wood composites’ mechanical and physical characteristics are influenced by a number of variables, including the mass ratio of the components, the amount of resin, the temperature, the pressure, and the duration of pressing [11,21]. Wood is heated to a high temperature and combined with other polymer components to create wood composites. The composite may become unstable at high temperatures, which would diminish its mechanical qualities [24]. The combination of wood particles and rubber granulate in particleboard could improve some physical or mechanical properties.

This research follows up on the issue of single-layer particleboards, where rubber granulates were added of the same fraction as mentioned in this article. The granulates were implemented into the particleboards in different weight proportions, namely 10%, 15%, and 20% [25]. As part of this research, many properties were carried out (mechanical, physical, acoustic, thermophysical, structural, fire-fighting, insulating, environmental). The main aim of the research was to use waste rubber and thus reduce the environmental burden and save primary raw materials by using waste. In the research about single-layer particle boards, the results show that increasing the proportion of granulate improved most of the properties. However, the problem was the reduction in mechanical properties. Therefore, the next step was to incorporate a 10% proportion into three-layer particleboard to improve mechanical properties. The purpose of this study was to assess the physical (density, water absorption, thickness swelling) and mechanical (internal bonding strength, modulus of rupture, modulus of elasticity, screw driving torque) characteristics of PBs containing waste rubber (tires, seals, carpets, and cables) in the proportion of 10% by weight in the core layer and to compare the findings with those of PB without rubber filler. This paper provides new insights into the properties of particleboard containing waste rubber materials from the automotive industry and electrical cables.

## 2. Materials and Methods

### 2.1. Rubber Material

**Figure 1:**
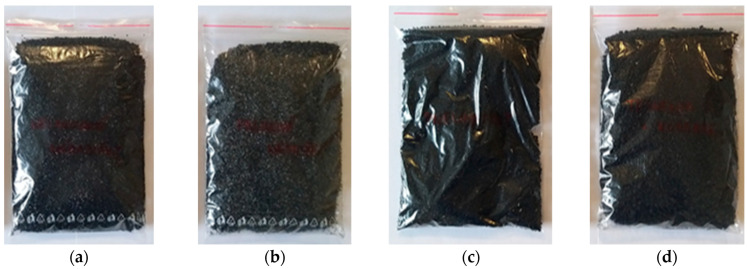
Waste rubber granulates: (**a**) tires; (**b**) seals and carpets; (**c**) external non-flammable cables; (**d**) internal flammable cables.

Granulates made from waste tires (WTs) consisting of styrene butadiene rubber (SBR), waste seals and carpets (WSCs) consisting of ethylene propylene diene monomer rubber (EPDM), waste external non-flammable cables (WENCs) consisting of fire-retardant non-corrosive rubber (FRNC), and waste internal flammable cables (WIFCs) consisting of cross-linked polyethylene (XLPE) were used as additional rubber. The size of the mentioned granulates was from 1.0 to 4.0 mm. WT and WSC granulates were produced by AVE SK plant in Kechnec (Perinska street 285, 044 58 Kechnec, Slovakia) and WENC and WIFC granulates were produced by the Faculty of Production and Power Engineering at the University of Agriculture in Krakow (Poland). Additional rubber granulates are represented by Figure 1. The main properties and descriptions of additional rubber granulates are listed in Table 1.

### 2.2. Particleboards Processing

In the three-layer PB processing, wood particles for surface and core layers were made from spruce non-treated beams at the Technical University in Zvolen. Spruce beams were obtained from wooden buildings after their life cycle in the Banská Bystrica region (Slovakia). The dimensions of particles were from 0.25 to 1.0 mm for the surface layer and from 0.25 to 4.0 mm for the core layer. Surface particles were dried to a moisture of 6% and core particles to a moisture of 4%.

A commercially available melamine–formaldehyde resin was used for surface particles, and urea–formaldehyde resin in combination with melamine–formaldehyde resin (3:1) was used for core particles. Paraffin emulsion (35%) as an excipient and ammonium nitrate NH_4_NO_3_ (47%) as a hardener was added to the adhesive mixtures. The quantities of used materials for 1 particleboard are listed in Table 2.

**Figure 2:**
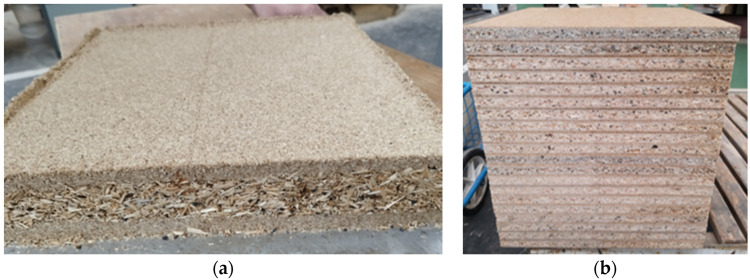
Particleboards processing: (**a**) mat formatting; (**b**) finished particleboards.

PBs with the addition of waste rubber (10% of the weight in the core layer) had the dimensions of 360 mm × 360 mm × 18 mm (Figure 2).

PBs were made using standard technology in the laboratories of the Technical University in Zvolen (Slovakia). Particle mats were first cold-pressed at 1 MPa, then hot-pressed in a laboratory press CBJ 100–11 (TOS, Rakovník, former Czechoslovakia) with a maximum temperature of 230 °C, a maximum pressing pressure of 5 MPa, and a total pressing time of 5 min [28], which had to be longer due to the presence of waste rubber granulate in the core layer. The complete methodology for the production of PBs was used from utility model no. 10249 [29]. Finally, 6 PBs of each condition (30 in total) were produced, as shown in Table 3.

### 2.3. Methodology of Physical Properties

Using the EN 323 (2002) [30] standard, the density of each sample was evaluated. Measurements were carried out on 6 samples per each type of PB according to the EN 326 (1994) [31]. The density was calculated by Equation (1):

(1) $$\rho =\frac{m}{b_{1}\times b_{2}\times t}(g\times {cm}^{-3})$$

where m is weight of the sample in grams, b_1_ is length of the sample in millimeters, b_2_ is width of the sample in millimeters, and t is thickness of the sample in millimeters.

According to the EN 317 (2005) [32] standard, water absorption (WA) and thickness swelling (TS) of each sample after 2 and 24 h were evaluated. Measurements were carried out on 8 samples per each type of PB according to the EN 326 (1994) [31]. The WA and TS were calculated by Equation (2):

(2) $$G_{t}=\frac{t_{2}-t_{1}}{t_{1}}\times 100(\%)$$

where t_2_ is the value of the sample after immersion in grams for WA and in millimeters for TS, and t_1_ is value of the sample before immersion in grams for WA and in millimeters for TS.

**Figure 3:**
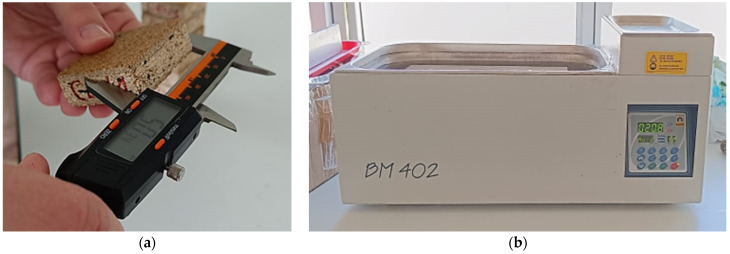
Physical properties: (**a**) methodology of density measurement; (**b**) methodology of water absorption and thickness swelling measurement.

The principle of this method is to place samples in water bath BM 402 (Nüve, Ankara, Turkey) and then measure their thickness and weight after 2 and 24 h. The methodology of physical properties is shown in the Figure 3. Analysis of variance—ANOVA (TIBCO Statistica^TM^ 14.0.0, TIBCO Software Inc., Palo Alto, CA, USA) was used for examination of differences in density, WA, and TS values.

### 2.4. Methodology of Mechanical Properties

According to the EN 319 (2005) [33] standard, the internal bonding strength (IBS) of each sample was evaluated. Measurements were carried out on 8 samples per each type of PB according to the EN 326 (1994) [31]. The IBS was calculated by Equation (3):

(3) $$f_{t}=\frac{F_{max}}{a\times b}(MPa)$$

where F_max_ is load acting on the sample at failure in newtons, a is length of the sample in millimeters, and b is width of the sample in millimeters.

The principle of this method is to pull the sample and record the IBS stress at the point of disruption afterward. A laboratory testing machine AG–IC (Shimadzu, Kyoto, Japan) was used for sample measurement.

According to the EN 310 (2005) [34] standard, the bending strength (BS), specifically modulus of rupture (MOR) and modulus of elasticity (MOE) of each sample, were evaluated. Measurements were carried out on 6 samples per each type of PB according to the EN 326 (1994) [31]. The MOR was calculated by Equation (4) and the MOE was calculated by Equation (5):

(4) $$f_{m}=\frac{3\times F_{max}\times l_{1}}{2\times b\times t^{2}}(MPa)$$

where F_max_ is breaking load in newtons, l_1_ is distance between the centers of the supports in millimeters, b is width of the sample in millimeters, and t is thickness of the sample in millimeters.

(5) $$E_{m}=\frac{l_{1}^{3}\times (F_{2}-F_{1})}{4\times b\times t^{3}\times (a_{2}-a_{1})}(MPa)$$

where l_1_ is distance between the centers of the supports in millimeters, F_2_ − F_1_ is load increase in newtons, b is width of the sample in millimeters, t is thickness of the sample in millimeters, and a_2_ − a_1_ is deflection increase at half the length of the sample in millimeters.

The principle is to load the sample and record the BS at break afterward. A laboratory testing machine AG–IC was used for samples measurement.

The screw driving torque (SDT) was also measured on the samples, namely seating torque (SeT) and stripping torque (StT). Measurements were carried out on 8 samples per each type of PB. The principle of the test is screwing the screw into the sample with subsequent recording of the torque when the screw is turning without resistance.

**Figure 4:**
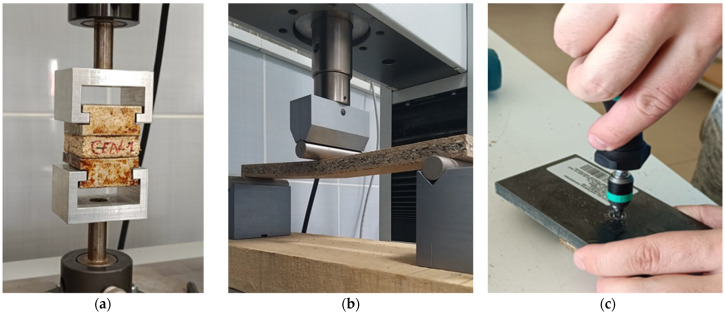
Mechanical properties: (**a**) methodology of internal bonding strength measurement; (**b**) methodology of bending strength measurement; (**c**) methodology of screw driving torque measurement.

The methodology of mechanical properties is shown in Figure 4. Analysis of variance—ANOVA was used to examine for differences in IBS, MOR, MOE, SeT, and StT values.

## 3. Results

### 3.1. Evaluation of Physical Properties

**Figure 5:**
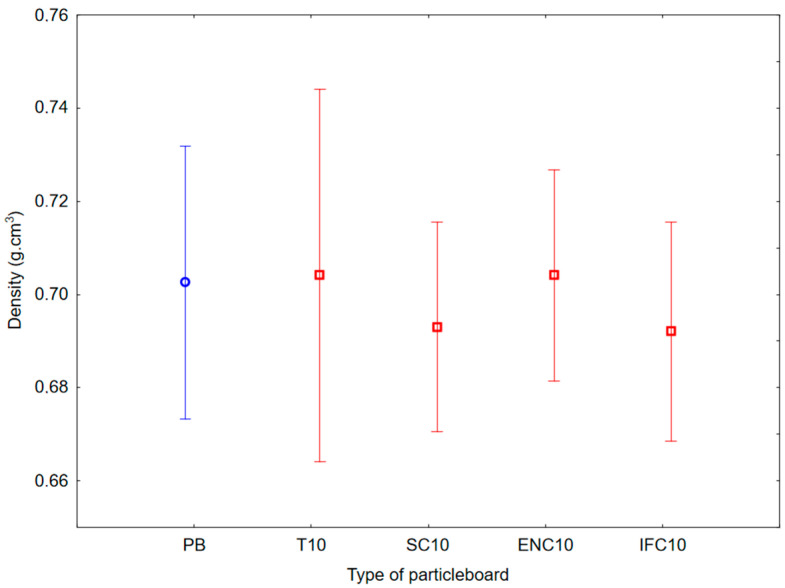
Graph of 95% confidence intervals for density of particleboards containing rubber filler: (PB) particleboard without rubber filler; (T10) particleboard containing 10% of waste tires; (SC10) particleboard containing 10% of waste seals and carpets; (ENC10) particleboard containing 10% of waste external non-flammable cables; (IFC10) particleboard containing 10% of waste internal flammable cables.

The average results of density values are shown in Figure 5. Based on the values, there is no significant difference and it can be concluded that the average density of each type of PB ranged from 0.692 to 0.704 g·cm^−3^. These values of density are typical for common single-layer and three-layer PBs.

ANOVA analysis of physical properties, namely WA and TS after 2 and 24 h, is shown in Table 4.

**Figure 6:**
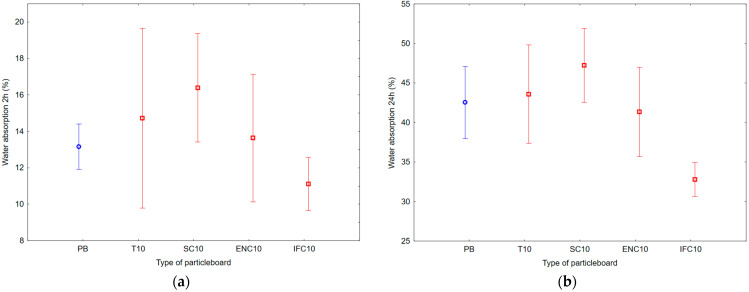
Graph of 95% confidence intervals for water absorption (WA) of particleboards containing rubber filler: (**a**) after 2 h; (**b**) after 24 h; (PB) particleboard without rubber filler; (T10) particleboard containing 10% of waste tires; (SC10) particleboard containing 10% of waste seals and carpets; (ENC10) particleboard containing 10% of waste external non-flammable cables; (IFC10) particleboard containing 10% of waste internal flammable cables.

**Figure 7:**
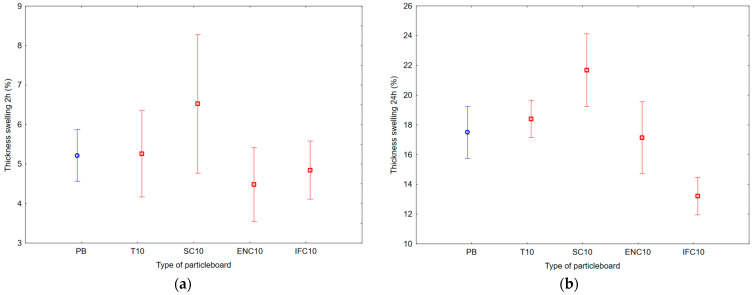
Graph of 95% confidence intervals for thickness swelling (TS) of particleboards containing rubber filler: (**a**) after 2 h; (**b**) after 24 h; (PB) particleboard without rubber filler; (T10) particleboard containing 10% of waste tires; (SC10) particleboard containing 10% of waste seals and carpets; (ENC10) particleboard containing 10% of waste external non-flammable cables; (IFC10) particleboard containing 10% of waste internal flammable cables.

As a result of ANOVA analysis, it is visible that there is a statistically significant difference in the groups of TS and WA after 24 h. However, there is not a statistically significant difference in the groups of TS and WA after 2 h. The results of Duncan’s analysis are shown in Table 5, which is represented by Figure 6 for WA results and Figure 7 for TS results.

Based on the values, it can be concluded that the average WA after 2 h ranged from 11.11 to 16.39%, and after 24 h, it ranged from 32.79 to 47.22%. The results show that the highest WA values had PB samples with the addition of WSC, and the lowest WA values had PB samples with the addition of WIFC.

Based on the values, it can be concluded that the average TS after 2 h ranged from 4.48 to 6.53%, and the average TS after 24 h ranged from 13.21 to 21.68%. The results show that the highest TS values had PB samples with the addition of WSC, and the lowest TS values had PB samples with the addition of WIFC.

### 3.2. Evaluation of Mechanical Properties

ANOVA analysis of mechanical properties, namely IBS, MOR, MOE, SeT, and StT, is shown in Table 6.

**Figure 8:**
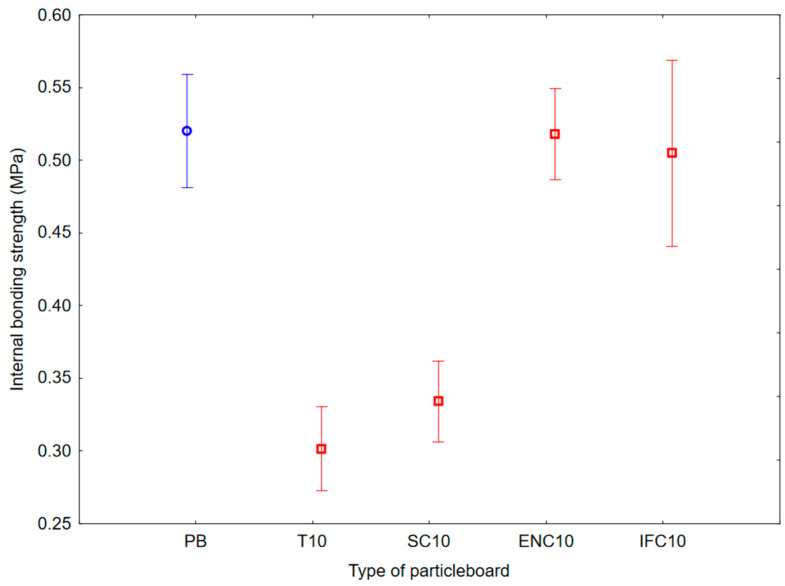
Graph of 95% confidence intervals for internal bonding strength (IBS) of particleboards containing rubber filler: (PB) particleboard without rubber filler; (T10) particleboard containing 10% of waste tires; (SC10) particleboard containing 10% of waste seals and carpets; (ENC10) particleboard containing 10% of waste external non-flammable cables; (IFC10) particleboard containing 10% of waste internal flammable cables.

**Figure 9:**
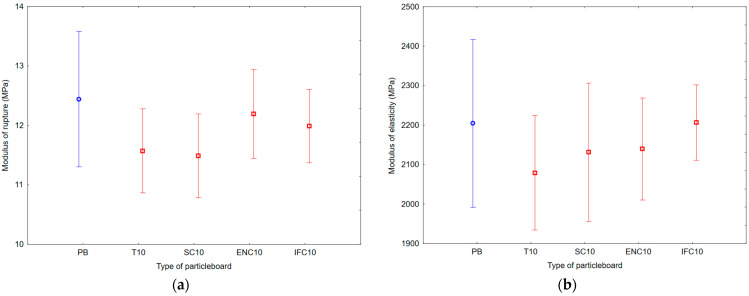
Graph of 95% confidence intervals for bending strength (BS) of particleboards containing rubber filler: (**a**) modulus of rupture; (**b**) modulus of elasticity; (PB) particleboard without rubber filler; (T10) particleboard containing 10% of waste tires; (SC10) particleboard containing 10% of waste seals and carpets; (ENC10) particleboard containing 10% of waste external non-flammable cables; (IFC10) particleboard containing 10% of waste internal flammable cables.

**Figure 10:**
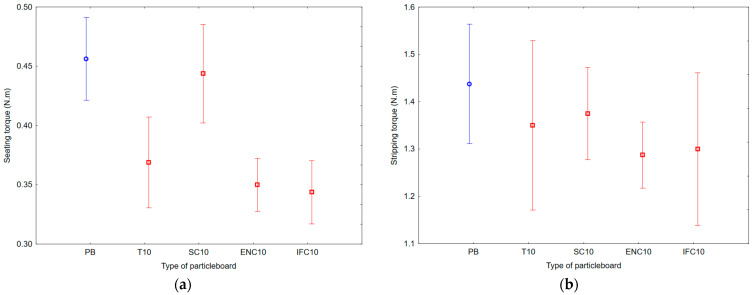
Graph of 95% confidence intervals for screw driving torque (SDT) of particleboards containing rubber filler: (**a**) seating torque; (**b**) stripping torque; (PB) particleboard without rubber filler; (T10) particleboard containing 10% of waste tires; (SC10) particleboard containing 10% of waste seals and carpets; (ENC10) particleboard containing 10% of waste external non-flammable cables; (IFC10) particleboard containing 10% of waste internal flammable cables.

As a result of ANOVA analysis, a statistically significant difference was found in the IBS and SeT groups, but no statistically significant difference was found in the MOR, MOE, and StT groups. The results of Duncan’s analysis are shown in Table 7, which is represented by Figure 8 for IBS results, Figure 9 for MOR and MOE results, and Figure 10 for SeT and StT results.

Based on the values, it can be concluded that the average IBS ranged from 0.30 to 0.52 MPa. The results show that the highest IBS values had PB samples with no addition of waste rubber, and the lowest IBS values had PB samples with addition of WT.

Based on the values, it can be concluded that the average MOR ranged from 11.49 to 12.44 MPa and average MOE ranged from 2079.07 to 2206.29 MPa. The results show that the highest MOR values had PB samples with no addition of waste rubber, the lowest MOR values had PB samples with addition of WSC, the highest MOE values had PB samples with addition of WIFC, and the lowest MOE values had PB samples with addition of WT.

Based on the results, it can be concluded that the average SeT ranged from 0.34 to 0.46 N·m and average StT ranged from 1.29 to 1.44 N·m. The results show that the highest SeT values had PB samples with no addition of waste rubber, the lowest SeT values had PB samples with addition of WIFC, the highest StT values had PB samples with no addition of waste rubber, and the lowest StT values had PB samples with addition of WENC.

## 4. Discussion

Kőse et al. [35] investigated lightweight three-layered PBs using waste rubber from summer and winter tires in ratios of 10%, 20%, 30%, and 40%. The results of PBs with addition of 10% showed comparable values to our samples at TS and worse values by about 60% at WA. Buyuksari et al. [36] evaluated the PBs manufactured from waste stone pine cones in ratios from 10% to 50%. The results of TS of PBs with an addition of 10% showed similar values, and the results of WA reached worse properties by approximately 20% to 30%. Kord et al. [37] evaluated the similar PBs manufactured from Canola straws. The WA results of PBs without filler reached about 5% and the TS about 7.5%, better values in comparison with our PBs without rubber filler. Durmaz [38] investigated wood plastic composites made of wood flour in ratios of 70% and HDPE in ratios of 30%. The average results of WA reached 43.9%, which is comparable to our PBs, and the average results of TS reached approximately 5% worse values in our case. The tire rubber filler could improve the WA of the PBs due to its hydrophobic properties [16]. In this context, it is thought that the WA percentages of the samples were the highest in the SC10 group due to the spongy material in the WSC rubber mixture. In contrast, the reason why the WA percentage is the lowest in the IFC10 group could be due to the fact that the cables are produced using XLPE without additives. The use of fire-retardant chemicals in internal flammable cables is likely the cause of the slightly higher WA and TS values after 24 h for the ENC10 compared to the IFC10. According to the literature, one of the most popular polymers for electrical and control cables is polyvinyl chloride, or PVC. Using inorganic or organic flame retardants as cable chemicals creates fireproof functionality.

Large quantities (60–70%) of inorganic filler materials such as metal hydroxides (aluminum trihydroxide or magnesium hydroxide) are widely used [39]. Also, these cables are sheathed with a halogen-free polyolefin [40]. From the point of view of water absorption and thickness swelling, the best results were evaluated for IFC10 samples. These composites could be used in spaces with higher humidity, e.g., bathrooms.

As mentioned before, Kőse et al. [35] analyzed lightweight three-layered PBs using waste rubber from summer and winter tires in various ratios of granulates. The results of PBs with an addition of 10% show lower values in comparison with our PBs, namely 0.26–0.33 MPa in IBS, 3.63–5.14 MPa in MOR, and 807.11–1100.33 MPa in MOE. Buyuksari et al. [36] found out that the mechanical properties of PBs with an addition of 10% showed 0.528–0.568 MPa values in IBS, 14.66–16.07 MPa values in MOR, and 1879.3–2068.6 MPa values in MOR. Based on the results, IBS values were comparable to our PB, ENC10, and IFC10 samples, MOR values were lower by about 2–4 MPa in our case, and MOE values were higher by about 100–200 MPa in our case. Comparable results for single-layer particleboards containing 10% of tires and a mixture of seals and carpets were obtained in another research study. Results of tensile strength for particleboards containing 10% of tires filler were lower by 27% compared to the reference samples [25]. The incorporation of waste rubber materials after their life cycle often leads to a decrease in mechanical properties due to insufficient interfacial interaction [19,41]. Regarding the tensile strength of SBR, the range is approximately 3.5 to 20 MPa. For elongation, the range is approximately 450 to 600%. Tensile strength of EPDM has a range of approximately 3.5 to 17 MPa. Elongation represents a range of approximately 100 to 700% [26]. For FRNC and XLPE, mechanical properties are not stated, since their application is primarily in electronics [27]. However, based on the results, it can be assumed that the mechanical properties will be higher than for SBR and EPDM. However, it should be noted that the used rubber materials were in the form of granulates.

Based on our results, comparable mechanical properties (except for SeT) with the reference particleboard were found only for ENC10 and IFC10. For other particleboards containing rubber filler (T10, SC10), we recorded lower mechanical strength values. It is thought that the spongy materials in the structure of the SC10 group have a decreasing effect on the IBS resistance due to its soft structure and low-strength properties. The lowest IBS resistance was observed in the T10 group, which could be due to the lack of sufficient bonding between the glue and the rubber and the strength-reducing effect of the soft structure of the rubber. Stress concentration occurs in rubber crumbs due to their lower strength compared to the wood particles. This stress concentration greatly reduces the IBS strength of the samples [16,42]. As a result of the soft structure and low-strength properties of the spongy materials in the structure of the SC10 group, the MOR and MOE values are thought to be reduced compared to the reference samples. Waste tire rubber has a Poisson’s ratio of 0.5, making it incompressible, and as a result, a decrease in bending strength values occurs in the samples. Similar results for wood fiber/waste tire rubber composites have been observed by Ayrilmis et al. [16]. Additionally, no significant differences were observed among the groups in terms of MOR and MOE. Analyzing the results, we found that SeT values of ENC10, IFC10, and T10 groups are very close to each other. Maximum values were observed in SC10 and PB groups. As an explanation for this situation, the density values of the polymers added to the ENC10, IFC10, and T10 groups are close to each other, and the cavity structure in the samples is also similar to each other. In the SC10 group, it is thought that the spongy structure causes the cavity structure in the samples to be tighter, and as a result, the SeT value is higher [43,44,45]. Considering the above results, it can be concluded that the most suitable materials in terms of the physical and mechanical properties performed are ENC10 and IFC10 samples. These PBs have the prerequisites to replace commercial particleboards and can be utilized in practice—for example, as a component of flooring, furniture, or insulation.

## 5. Conclusions

The article’s aim was to evaluate novel rubber-containing particleboards made from waste materials, which positively contributes to environmental protection, saving primary resources and reducing production costs. This work assessed the influence of the type of rubber filler (tires, mixture of seals and carpets, non-flammable cables, flammable cables) added in a volume of 10% to the middle layer of three-layer particleboards (PBs) on its physical and mechanical properties. Based on the results of physical properties, it can be stated that the density of the prepared PBs containing rubber filler was comparable to reference PBs; similar results for water absorption (WA) and thickness swelling (TS) were achieved for sample PBs, PBs containing waste tires (T10), PBs containing waste seals and carpets (SC10), and PBs containing waste external non-flammable cables (ENC10). The lowest values were obtained for PBs containing waste internal flammable cables (IFC10), probably due to the kind of rubber filler used, which was based on cross-linked polyethylene. Due to the lowest WA and TS, it is therefore appropriate to also use these composites in areas with higher humidity.

Based on the results for mechanical properties, it can be stated that comparable results for internal bonding strength (IBS) were found out for PB, ENC10, and IFC10 samples. Values of IBS for T10 and SC10 samples were lower by more than 40% compared to reference samples, probably due to the type of rubber filler. There are spongy materials in the structure of the SC10 samples, which has a decreasing effect on the IBS resistance; in the case of the T10 samples, it was due to the lack of sufficient bonding between the glue and the rubber and the strength-reducing effect of the soft structure of the rubber. No significant changes were observed in modulus of rupture (MOR) and modulus of elasticity (MOE) properties. Seating torque (SeT) values of the SC10 samples are close to the values of the reference samples. In this context, the PBs produced with ethylene propylene diene monomer rubber in areas requiring screw-holding strength are another significant result in adding value to the waste material.

Based on this issue of wood–rubber composites, the question is how to recycle the mentioned particleboards with rubber filler. The most realistic way would be to crush the composites and separate the individual materials from each other using physical and chemical processes. The separated materials can then be reused. The second way could be energy recovery, which should, however, be avoided, as it is not beneficial for the environment.

## Figures and Tables

**Table 1:** Description of used rubber materials.

Granulate	Material	Main Properties	Size (mm)	Bulk Density (g·cm^−3^)
Tires	SBR	abrasion resistance, adhesion to metal and rigid materials, compression set, impact resistance, tear resistance, water and oxidation resistance [26]	1.0 to 4.0	0.43
Seals and carpets	EPDM	adhesion to metal and rigid materials, compression set, chemical resistance, colorability, environmental resistance [26]	1.0 to 4.0	0.37
External non-flammable cables	FRNC	flexibility, low-smoke, zero-halogen, flame-retardant, heat resistance, isolation, temperature range [27]	1.0 to 4.0	0.42
Internal flammable cables	XLPE	flexibility, low-smoke, zero-halogen, isolation, temperature range [27]	1.0 to 4.0	0.41

**Table 2:** Quantities of used materials for 1 particleboard.

Layer	Wood Particles (g)	Resin (g)	Paraffin Emulsion (g)	Ammonium Nitrate (g)	Granulate (g)
Surface	670	103.9	14.5	4.4	0
Core	888.3	99.1	16.3	8.4	98.7

**Table 3:** Signification of samples.

Sample	Characterization
PB	Particleboard without rubber filler
T10	Particleboard containing 10% of WTs
SC10	Particleboard containing 10% of WSCs
ENC10	Particleboard containing 10% of WENCs
IFC10	Particleboard containing 10% of WIFCs

(WTs) waste tires; (WSCs) waste seals and carpets; (WENCs) waste external non-flammable cables; (WIFCs) waste internal flammable cables.

**Table 4:** ANOVA analysis of water absorption (WA) and thickness swelling (TS) of particleboards containing rubber filler.

Variance Analysis	Mean Square	F	Sig.
TS after 2 h	29.567	1.907	0.134 *
TS after 24 h	73.926	14.916	0.000
WA after 2 h	4.911	2.63	0.053 *
WA after 24 h	218.064	44.694	0.000

*: There is no significant difference.

**Table 5:** Duncan’s analysis of water absorption (WA) and thickness swelling (TS) of particleboards containing rubber filler.

Sample	WA (%)		TS (%)	
	2 h	24 h	2 h	24 h
PB	13.16 * ± 1.48	42.53 ^a^ ± 5.44	5.22 * ± 0.78	17.50 ^b^ ± 2.08
T10	14.73 * ± 5.90	43.58 ^a^ ± 7.46	5.26 * ± 1.31	18.40 ^b^ ± 1.49
SC10	16.39 * ± 3.56	47.22 ^a^ ± 5.61	6.53 * ± 2.10	21.68 ^a^ ± 2.92
ENC10	13.63 * ± 4.19	41.35 ^a^ ± 6.75	4.48 * ± 1.11	17.13 ^b^ ± 2.90
IFC10	11.11 * ± 1.74	32.79 ^b^ ± 2.58	4.85 * ± 0.88	13.21 ^c^ ± 1.52

Note: Different letters indicate different homogeneity groups (*p* ≤ 0.05). *: There is no significant difference. (PB) particleboard without rubber filler; (T10) particleboard containing 10% of waste tires; (SC10) particleboard containing 10% of waste seals and carpets; (ENC10) particleboard containing 10% of waste external non-flammable cables; (IFC10) particleboard containing 10% of waste internal flammable cables.

**Table 6:** ANOVA analysis of internal bonding strength (IBS), modulus of rupture (MOR), modulus of elasticity (MOE), seating torque (SeT), and stripping torque (StT) of particleboards containing rubber filler.

Variance Analysis	Mean Square	F	Sig.
IBS	0.076	13.675	0.000
MOR	1.578	1.533	0.222 *
MOE	29,916.94	0.932	0.461 *
SeT	0.019	11.733	0.000
StT	0.019	0.661	0.624 *

*: There is no significant difference.

**Table 7:** Duncan’s analysis of internal bonding strength (IBS), modulus of rupture (MOR), modulus of elasticity (MOE), seating torque (SeT), and stripping torque (StT) of particleboards containing rubber filler.

Sample	IBS (MPa)	MOR (MPa)	MOE (MPa)	SDT (N·m)	
				SeT	StT
PB	0.52 ^a^ ± 0.05	12.44 * ± 1.08	2204.39 * ± 202.46	0.46 ^a^ ± 0.042	1.44 ^a^ ± 0.05
T10	0.30 ^b^ ± 0.03	11.57 * ± 0.67	2079.07 * ± 137.94	0.37 ^b^ ± 0.046	1.35 ^b^ ± 0.03
SC10	0.33 ^b^ ± 0.03	11.49 * ± 0.67	2131.36 * ± 167.02	0.44 ^a^ ± 0.050	1.38 ^a^ ± 0.03
ENC10	0.52 ^a^ ± 0.04	12.19 * ± 0.71	2139.71 * ± 123.01	0.35 ^b^ ± 0.027	1.29 ^b^ ± 0.04
IFC10	0.50 ^a^ ± 0.08	11.99 * ± 0.59	2206.29 * ± 90.69	0.34 ^b^ ± 0.032	1.30 ^b^ ± 0.08

Note: Different letters indicate different homogeneity groups (*p* ≤ 0.05). *: There is no significant difference. (PB) particleboard without rubber filler; (T10) particleboard containing 10% of waste tires; (SC10) particleboard containing 10% of waste seals and carpets; (ENC10) particleboard containing 10% of waste external non-flammable cables; (IFC10) particleboard containing 10% of waste internal flammable cables.

## Data Availability

The data presented in this study are available on request from the corresponding author.

## Abbreviations

The following abbreviations are used in this manuscript:

SBR	Styrene butadiene rubber
EPDM	Ethylene propylene diene monomer rubber
FRNC	Fire retardant non-corrosive rubber
XLPE	Cross-linked polyethylene
PB	Particleboard without rubber filler
WTs	Waste tires
WSCs	Waste seals and carpets
WENCs	Waste external non-flammable cables
WIFCs	Waste internal flammable cables
T10	Particleboard containing 10% of waste tires
SC10	Particleboard containing 10% of waste seals and carpets
ENC10	Particleboard containing 10% of waste external non-flammable cables
IFC10	Particleboard containing 10% of waste internal flammable cables
WA	Water absorption
TS	Thickness swelling
IBS	Internal bonding strength
BS	Bending strength
MOR	Modulus of rupture
MOE	Modulus of elasticity
SDT	Screw driving torque
SeT	Seating torque
StT	Stripping torque
